# Uterus Transplantation from Deceased Donors: First Italian Experience

**DOI:** 10.3390/jcm13226821

**Published:** 2024-11-13

**Authors:** Pierfrancesco Veroux, Paolo Scollo, Alessia Giaquinta, Giuseppe Roscitano, Martina Maria Giambra, Basilio Pecorino, Concetta De Pasquale, Giuseppe Scibilia, Maria Luisa Pistorio, Massimiliano Veroux

**Affiliations:** 1 Vascular Surgery and Organ Transplant Unit, University Hospital of Catania, 95121 Catania, Italy; pveroux@unict.it (P.V.); alessiagiaquinta@gmail.com (A.G.); giuseppe.roscitano@virgilio.it (G.R.); giambramartina@gmail.com (M.M.G.); depasqua@unict.it (C.D.P.); marialuisa.pistorio@unict.it (M.L.P.); 2 Maternal and Child Department, Obstetrics and Gynecology, Cannizzaro Hospital, 95126 Catania, Italy; paolo.scollo@unikore.it (P.S.); basilio.pecorino@unikore.it (B.P.); 3 Obstetrics and Gynecology Giovanni Paolo II Hospital, 97100 Ragusa, Italy; g.scibilia@libero.it

**Keywords:** Mayer–Rokitansky–Küster–Hauser syndrome, acute rejection, donor procurement, graft loss, hysterectomy, live births, deceased donor

## Abstract

**Background**: Uterus transplantation (UTx) is currently the only available treatment for absolute uterine factor infertility. Deceased donors have recently emerged as a valid alternative to living donors for uterus transplantation, with similar results. **Methods**: We report the first experience in Italy of uterus transplantation from deceased donors. Three uterus transplantations from deceased donors were performed at the Organ Transplant Unit of the University Hospital of Catania, Italy, between August 2020 and January 2022. **Results**: Two patients underwent UTx due to Mayer–Rokitansky–Küster–Hauser syndrome, while one patient had a previous hysterectomy due to benign disease. The donors’ ages were between 25 and 43 years and the mean cold ischemia time was 18.3 h. The mean age of the recipients was 31.6 years, and the mean recipient surgery duration was 5.3 h, with a mean blood loss of 766.66 mL. Two recipients developed a post-transplant hematoma, which was treated conservatively. No uterus recipient needed a re-operation during the first 30 days after transplantation. No histological signs of acute rejection were detected at the cervical biopsies performed at 1, 3, and 6 months after transplantation. First menstruation occurred in all recipients after 39 ± 12 days after transplantation. One live birth was reported 15 months after UTx. One graft was lost three months after UTx due to graft thrombosis. **Conclusions**: Uterus transplantation from deceased donors is emerging as a valid alternative to living donors in order to increase the donor pool.

## 1. Introduction

There is an increasing interest in uterus transplantation (UTx) as an alternative to gestational surrogacy and adoption for patients with uterine factor infertility (UFI), related either to iatrogenic causes (e.g., hysterectomy for benign disease, post-partum bleeding, or Asherman’s syndrome) or congenital causes (uterine agenesis in Mayer–Rokitansky–Küster–Hauser (MRKH) syndrome or partial uterine malformation) [1,2,3,4,5]. Uterus transplantation is the result of many years of extensive foundational work with a wide range of animal species including higher primates [3]. It is intended to restore anatomical normalcy in women with UFI, giving them the possibility of carrying their own pregnancy and delivering children [6]. The first successful uterus transplantation from a deceased donor was performed in Turkey [7,8], immediately followed by the first successful live birth after uterus transplantation from a living donor [9]. Many reports have been published in the literature on uterus transplantation activity, but recent reports from the Registry of the International Society of Uterus Transplantation and from the third International Congress of the International Society of Uterus Transplantation have described a total of 91 UTx procedures performed worldwide [2,6,7,8,9,10,11]. However, living donor hysterectomy is a challenging procedure, with a potentially increased rate of complications compared to conventional hysterectomy. This has generated many ethical concerns about the opportunity of using a living donor for UTx, and thereby raised an increased interest in UTx from deceased donors [12,13,14]. In 2018, the Italian Institute of Health approved the first experimental protocol for uterus transplantation from deceased donors, and this study presents the results of the first three UTx procedures performed.

## 2. Material and Methods

The experimental protocol of human UTx from deceased donors in Italy was approved in 2018 by the Italian Institute of Health, the National Transplant Centre, the University of Catania, the Cannizzaro Hospital, and the Policlinico San Marco University Hospital Medical Research Committee (No. 1438/CNT2018) [15]. The clinical group involved in the experimental study of UTx involved many professionals with a specific experience in solid organ transplantation (kidney transplantation from either living and deceased donors and pancreas transplantation) as well as vascular surgery and oncological gynecologic surgery. Moreover, many other professionals from reproductive endocrinology, maternal–fetal medicine, anesthesia, psychiatry, infectious disease, and neonatology were included.

Donor inclusion criteria were heart-beating brain-dead donors without ongoing infective and/or neoplastic risk (standard donor), age >18 and <45 years, no ongoing pregnancy, no previous Caesarian section, and no previous uterine diseases altering the structural suitability of the uterus (no leiomyomas or uterine anomalies based on ultrasonography and/or computed tomography and no previous uterine surgery). Donor evaluation also excluded active infections (gonorrhea, chlamydia, syphilis, Hepatitis B and C, HIV, and HTLV). Moreover, multi-organ donors were matched for blood type and should have ≥2 HLA matches with the recipient.

Women with UFI could be scheduled for uterus transplantation based on the following inclusion criteria:

- Pre-menopausal age
- Uterine agenesis (MRKH syndrome or Asherman’s syndrome) or hysterectomy for benign disease or partial uterine malformation
- Absence of co-morbidities contraindicating the uterus transplant (diabetes, cardiovascular or pulmonary disease, active infections or cancer)
- Signed a detailed informed consent highlighting the risk and potential benefits of uterus transplantation
- Complete psychological and psychiatric evaluation aiming to evaluate the motivation of the potential recipient

### 2.1. Surgical Technique

This study reported the procurement of the first 4 uteri from brain-dead multi-organ donors in Italy between August 2020 and January 2022. Since this was the only protocol approved for uterus transplantation in Italy, potential recipients came from all regions of Italy and, to minimize the cold ischemia time, the potential recipient was immediately called when a potential uterus donor was available. After evaluating the suitability of the procured uterus, based on adequate perfusion or absence of vascular lesions, the bench surgery was then performed at the Organ Transplant Unit of the University Hospital of Catania. All surgeries were performed in agreement with other abdominal transplant teams, and were adapted to the need of the different teams. The uterus was procured after all vital organs.

#### 2.1.1. Donor Surgery

Before proceeding to organ procurement, a vigorous vaginal antisepsis with 10% iodopovidone solution was performed. No modifications of donor position or preparation were needed for uterus procurement. However, during the warm phase, the two femoral arteries were dissected using two inguinal incisions. This allowed the positioning of a 12 Fr femoral introducer sheet for each femoral artery for the uterus perfusion during the cold phase of the organ procurement procedure after aortic cross-clamping. The surgical procedure began with a standard midline incision from the pubis to the sternotomy to expose all thoracic and abdominal organs. After the surgical teams were ready with all the vital organs (heart, lungs, liver, kidneys, pancreas, and intestine), the round ligament was transected as close as possible to the pelvic wall. Then, the pelvic peritoneum was incised, and the retroperitoneum was prepared to develop the paravesical and pararectal spaces. The ovaries and fallopian tubes were separated from the uterus and left in place. The vesicocervical and rectovaginal spaces were dissected well below the vaginal fornix [13,16]. The common and internal iliac arteries were gently dissected to identify the uterine arteries at their origin from the internal iliac artery. This step was particularly important as it was mandatory to preserve an adequate patch of internal iliac arteries, including the uterine arteries of adequate length, for a safe transplant anastomosis. Iliac artery grafts can also be used for other vital organs, particularly pancreas and liver transplantation, so it was extremely important to have an agreement with the other transplant teams on the length and quality of the provided grafts. This step may be particularly conflicting, since vascular grafts were usually procured for liver or pancreas grafts and, in some cases, obtaining a vascular graft of sufficient length for uterus transplantation was not easy. Common and internal iliac veins were also gently dissected during the warm phase. After bilateral dissection, the sacral branches of the posterior and anterior divisions of the internal iliac artery were ligated, leaving only the patent uterine arteries [13]. Systemic heparinization with 3 mg/kg was then performed, and immediately after aortic cross-clamping, perfusion with a total of 4000 mL of Celsior^©^ (Genzyme Europe B.V. Gooimeer 10 NL-1411 DD, Naarden, The Netherland) solution was started and continued throughout all of the cold ischemia phase and concluded after uterus procurement. Once the procurement of the vital organs (including kidneys) was completed, the internal iliac vessels were cut above their anterior division and the umbilical, bladder, and vaginal vessels were cut below the parametria [16]. Finally, the gonadal vessels were cut as long as possible, and the ureters were dissected at their distal segment, in accordance with the kidney transplant team. At the end of the procedure, the explanted specimen consisted of the uterus and proximal vagina with the parametria attached to the anterior internal iliac vessels. The uterus was then packed in a cold preservation solution and ice and transferred to the organ transplant unit.

#### 2.1.2. Bench Surgery

The aim of the bench surgery was to evaluate the uterine vasculature and test the venous outflow. The first step included the ligation of the branch of the uterine arteries and veins with polypropylene 6/0. Then, the internal iliac patch was prepared, and the uterine vein was eventually elongated with a graft from the ovarian vein. This is easier when the kidney is procured by the same team, as it was in our case. Finally, the uterine veins were tested, and the uterine arteries were tested by infusing 100–200 mL of Celsior^©^ solution. The result was considered positive if the solution was seen exiting the gonadal and uterine veins [13]. One uterus graft was discarded due to insufficient venous drainage, despite the fact that elongation with an ovarian vein graft was attempted during bench surgery.

#### 2.1.3. Recipient Surgery

Recipient surgery began with a midline incision, and the removal of the rudimentary uterus (in two patients), with the clearance of the vaginal vault from the bladder. The recipient external iliac vessels were dissected. Uterine veins and/or utero-ovarian veins were anastomosed end-to-side with the external iliac vein on both sides with polypropylene 6/0, while the uterine artery with the iliac patch was anastomosed end-to-side to the external iliac artery on both sides with polypropylene 6/0. In two patients, the right uterine vein was elongated with an ovarian vein graft from the same donor (kidney transplantation performed by the same transplant team). After vascular declamping, a careful hemostasis of uterine vessels was performed, with ligation of all collaterals. The vault was opened and vaginal–vaginal anastomosis was accomplished. Fixation sutures were used to connect the round and uterosacral ligaments. At the end of the procedure, an indocyanine green fluorescence was performed in all recipients (Video S1). Finally, an echo color doppler of uterine vessels was performed before wound closure (Video S2). All recipients were recovered in a dedicated postoperative care unit for 3 days before being transferred to the ward.

### 2.2. Immunosuppression

All patients received an induction protocol with thymoglobulin at 1.5 mg/kg/die for 6 days, and steroids at a dose of 750 mg of prednisolone at the time of transplant and then at a dose of 1 mg/kg per day, which was slowly tapered to a maintenance dose of 5 mg/day by the end of the sixth month. Mycophenolate mofetil (MMF) was given at a dose of 1 to 2 g/day. Tacrolimus was initiated at 0.1 mg/kg/die, and doses were adjusted to keep levels between 10 and 12 ng/mL in the first month post-transplant and then between 6 and 8 ng/mL. The mycophenolate mofetil was replaced with azathioprine 50 mg/die 4 months after the transplantation. After surgery, the patients underwent rejection monitoring and uterine trophism, evaluated by cervical biopsies (Video S3), pelvic ultra-sound with doppler of the uterine vessels, and hormone dosages every 15 days for the first 3 months and then once a month for the next 3 months [15].

## 3. Results

A total of three uterus transplantations were performed between August 2020 and January 2022 (Table 1).

Two patients required uterus transplantation due to MRKH syndrome, while one patient received UTx due to a previous hysterectomy for benign disease. The donors’ ages were between 25 and 43 years, and in most cases they were multi-organ donors with most of their vital organs procured. In all three cases, the liver, pancreas, and kidneys were procured, and in one donor, a split liver procurement was performed. Since 2 of the 3 donors were not local, the cold ischemia time was long (mean 18.3 h). The mean recipient’s age was 31.6 years. All of the recipients were in good health at the time of the UTx, had a good histocompatibility (at least 2 HLA matches), and did not report significant co-morbidities. The mean recipient surgery duration was 5.3 h, and the mean blood loss was 766.66 mL. Two recipients developed a post-transplant hematoma, which was treated conservatively. None of the uterus recipients needed a re-operation during the first 30 days after transplantation. No histological signs of acute rejection were detected in the cervical biopsies performed 1, 3, and 6 months after transplantation. The first menstruation occurred in all recipients after 39 ± 12 days after transplantation. In patient 1, all of the cryopreserved oocytes were thawed and inseminated, with a 100% fertilization rate (6 fertilized oocytes out of the 6 available), as previously described [15]. At 15 months after transplantation a single vitrified and thawed blastocyst was transferred, achieving a viable pregnancy, which was complicated by a SARS-CoV-2 infection at week 30. The patient was admitted into the gynecology unit and an emergency Cesarean section was performed at 34 weeks of gestation because of fever and the appearance of regular uterine contractions. The patient delivered a female live birth, weighed 1725 g [15], who is now in good health 18 months after birth. Patient 2 was admitted two months after transplantation for severe respiratory distress. A naso-pharyngeal swab for SARS-CoV-2 was reported negative. A computed tomography (CT) chest scan demonstrated a diffuse bilateral ground glass opacity characterized by a partial consolidative pattern in the dorsal segments of the lower lobes. The patient was therefore admitted to the Intensive Care Unit and bronchoalveolar lavage demonstrated the presence of nucleic acids of *E-Aspergillus* and *E-Pneumocystis* DNA. The patient was immediately treated with Trimethoprim/Sulfamethoxazole 400 mg/5 mL + 80 mg/5 mL, Amphotericin B, and Imipenem/Cilastatin. After seven days the symptoms gradually improved, and a chest CT scan performed 25 days after the onset of the symptoms onset demonstrated the near complete resolution of pneumonia. We can report that the patient is in good health as of the 21 month post-transplant follow-up. Patient 3 required a graft hysterectomy three months after transplantation for graft thrombosis. No signs of acute rejection were detected during histological examination of the explanted uterus. Two patients developed a vaginal stenosis which was corrected using an off-label stent positioning in both cases.

## 4. Discussion

Uterus transplantation is unique in the field of solid organ transplantation, since it offers the extraordinary possibility to restore anatomical normalcy in women with UFI, giving them the possibility of having their own pregnancy and delivering a healthy child. This study reported the first Italian experience of uterus transplantation from a deceased donor, which is a valid alternative to living donors. To date, 25 uterus transplantations have been reported, resulting in 12 live births (a birth rate of 50%) [17], and nearly all transplants were performed in patients with MRKH syndrome [2,6,7,8,9,10,11,17]. Living donor UTx is a challenging and time-consuming procedure with higher hysterectomy-related risks compared to conventional surgical procedure [11]. These surgical issues, together with increasing ethical issues, raised concerns of an increasing utilization of deceased donors. While in principle, living uterus donors take the advantage of a more extensive evaluation of donors (particularly for uterus vasculature) with a potential better post-transplant outcome, it faces off with a challenging surgical procedure with potential life-threatening complications [11]. In this way, deceased donors may be a valid alternative, but only 8.5% of all deceased donors are finally considered potentially suitable for uterus transplantation [6,18]. The uterus is not a life-saving organ, and its procurement is usually delayed until the vital organs have been procured, as reported in our experience. However, some studies have reported that during multi-organ procurement, the uterus is prepared early and the removal occurs either prior to the retrieval of the other solid organs, or last [19]. However, the procurement of a uterus from a deceased donor could be faster and simpler than LDs, and the procurement of uterus vasculature may be easier, although many conflicts may arise with vital organ procurement (particularly the liver and pancreas). In our experience, all deceased donors with uterus donation were multi-organ donors, and iliac vessels were shared with surgical equipes for liver and pancreas procurement. This may raise some concerns about the length of the uterine artery and the internal iliac patch, which in many cases may be shared with other vital organs. Moreover, procurement of the uterine vein during cold ischemia time may be challenging and may increase the risk of non-utilization of the uterus. This was reported in our experience, where one graft was not used due to insufficient venous drainage. Uterus transplantation from deceased donors is affected by a longer cold ischemia time, although the safe tolerated cold ischemia time of the uterus is not yet known [9]. Finally, a careful dissection with ligation of the microvessels located in tissues surrounding the uterus is mandatory during procurement and bench surgery to prevent post-reperfusion bleeding in the recipient, although a similar rate of post-reperfusion bleeding complications between UTx from LDs and DDs has been reported [14].

One graft was lost in our experience: one patient required an early graft hysterectomy for graft thrombosis. This was related to an unsatisfactory graft re-perfusion, which probably caused an extensive ischemia-reperfusion injury leading to an early graft thrombosis. Graft failure has been reported with an incidence of 25% [17] and is mainly related to venous thrombosis. This highlights the need for a careful surgical technique in uterus procurement, particularly from deceased donors, to obtain an uterus vasculature of sufficient length: some authors [11,17] proposed to use ovarian veins in order to obtain a longer vein, and this could result in better outcomes and increase the rate of utilization of uteruses from deceased donors [17,19]. A late complication of UTx, which typically occurs several months after UTx, is the stenosis of the vaginal–vaginal anastomosis. This can affect up to 72% of recipients [17,19], make rejection monitoring difficult, and delay embryo transfer; it also limits the outflow of menstrual blood and sexual satisfaction [20]. Surgical repair and/or surgical dilatation with stent placement have been described in literature, with conflicting results [17]. Two patients in our experience had a vaginal stricture, successfully treated with a stent placement, which may be a valid alternative to surgery in selected cases [17]. A total of 12 live births have been reported in the literature after uterus transplantation from deceased donors, with a live birth rate of 66% [17]; we have previously described our first live birth in our three uterus transplantations, which was the first case in the world that was obtained from cryopreserved oocyte [17].

## 5. Conclusions

Uterus transplantation represents a promising treatment for women with uterine factor infertility. While most uterus transplantations in the world have been performed from living donors, uterus transplantation from deceased donors is emerging as a valid alternative. The increase in the donor pool will probably provide for an adequate number of viable grafts for transplantation, thus increasing the experience and safety of uterus transplantation from deceased donors, so that UTx from living donors would be no more ethically justifiable.

## Figures and Tables

**Table 1:** Uterus transplant recipients’ and donors’ characteristics.

Patient	Recipient Age (Years)	Indication for Transplant	Recipient BMI (kg/m^2^)	Waiting Time (Months)	HLA Match	Donor Age	Cold Ischemia Time (h)	Uterus Procurement Time (Warm + Cold Phases, min)	Surgical Time (h)	Blood Loss (mL)	Postoperative (30-Day) Complication	Length of Stay (Days)
1	32	MRKH	28	6	3	38	18	120	5.2	700	Peri-graft hematoma	20
2	34	MRKH	26	8	3	43	19	110	5.4	700	*Pneumocystis jirovecii* pneumonia	20
3	29	Hysterectomy for benign disease	26	6	3	25	18	120	6.1	900	Peri-graft hematoma	33

Legend: MRKH, Mayer–Rokitansky–Küster–Hauser syndrome; BMI, body mass index.

## Data Availability

It is possible for de-identified data to be made available upon reasonable request.

## Supplementary Materials

The following supporting information can be downloaded at: https://www.mdpi.com/article/10.3390/jcm13226821/s1, Video S1: Indocyanine green fluorescence imaging of the transplanted uterus demonstrated a correct perfusion of the graft; Video S2: The echo color doppler of uterus vasculature demonstrated a normal patency of uterine arteries and vein; Video S3: Cervical biopsy on a transplanted uterus.
